# Physical Activity in Children and Young Adults With Cerebral Palsy: Results From a Three‐Month Exercise Intervention

**DOI:** 10.1002/ejsc.12313

**Published:** 2025-04-30

**Authors:** Tiina Savikangas, Pedro Valadão, Eero A. Haapala, Iida Laatikainen‐Raussi, Timo Rantalainen, Taija Finni

**Affiliations:** ^1^ Faculty of Sport and Health Sciences University of Jyväskylä Jyväskylä Finland; ^2^ Neuromuscular Research Center Faculty of Sport and Health Sciences University of Jyväskylä Jyväskylä Finland; ^3^ Institute of Biomedicine School of Medicine University of Eastern Finland Kuopio Finland

## Abstract

Individuals with cerebral palsy (CP) are suggested to be less active than typically developing controls (TDs). However, their higher physical activity (PA) energy expenditure during a given activity should be considered. We investigated PA in 10 children and young adults with CP (seven males, 15.4 ± 5.1 years, and Gross Motor Function Classification System [GMFCS] level I/III) and eight TDs (six males, 15.3 ± 4.2 years). PA was assessed with a triaxial accelerometer at baseline and, in participants with CP, during a 3‐month exercise intervention. Data were analyzed for sedentary time, light PA, and moderate‐to‐vigorous PA using both general and, for participants with CP, GMFCS‐specific cutoffs. Statistical analyses included independent and paired sample *t*‐tests. Based on the general cutoffs, TD and CP participants accumulated on average 516.0 ± 122 and 610 ± 92 min/day sedentary time, 155 ± 37 and 138 ± 46 min/day light PA, and 76 ± 28 and 58 ± 30 min/day moderate‐to‐vigorous PA at baseline. No statistically significant between‐group differences were found (*p* ≥ 0.081). When applying GMFCS‐specific cutoffs, participants with CP on average accumulated 537 ± 105, 206 ± 61, and 64 ± 31 min/day sedentary time, light PA, and moderate‐to‐vigorous PA. These values differed from their values based on general cutoffs (*p* ≤ 0.004). The 51 min difference in light PA in favor of the CP group was borderline significant (*p* = 0.054). In the CP group, PA did not change from baseline to follow‐up (*p* ≥ 0.098). In conclusion, children and young adults with CP may be as active as TDs, especially when their higher PA energy cost is considered. Muscle strength and gait performance improving exercise did not seem promising in facilitating PA.

**Trial Registration:**

ISRCTN69044459

**Summary**

- We applied two raw accelerometry data processing approaches to investigate daily physical activity in children and young adults with and without cerebral palsy (CP). The raw accelerometry data processing approach choice proved highly relevant in participants with CP, whose physical activity energy cost is increased due to compromised movement efficiency.
- In participants with CP, using gross motor functioning‐specific physical activity intensity category cutoffs yielded 73 min less daily sedentary time, 67 min more light physical activity, and 6 min more moderate‐to‐vigorous physical activity per day compared to applying the general physical activity intensity cutoffs for adolescents.
- When applying the gross motor functioning‐specific cutoffs, individuals with CP accumulated on average 51 min more light physical activity than typically developing controls. When using the general cutoffs, statistically significant between‐group differences were not observed in any activity intensity.
- The muscle strength enhancing and gait performance improving EXECP intervention, which comprised strength, gait, and flexibility training, did not seem promising in facilitating daily physical activity.

## 1 Introduction

Physical activity (PA) is crucial for health and functioning across the lifespan (Bull et al. 2020; Chaput et al. 2020; Verschuren et al. 2016). Despite the numerous and well‐known benefits, insufficient PA is common in both adolescents and adults (Guthold et al. 2018, 2020). Alarmingly, individuals with cerebral palsy (CP) tend to be even less active than their typically developing peers from childhood to adulthood (Capio et al. 2012; Obeid et al. 2014; Okur et al. 2021; Nooijen et al. 2014; Aviram et al. 2019; Nieuwenhuijsen et al. 2009). CP describes a group of permanent disorders of the development of movement and posture, which causes activity limitations (Rosenbaum et al. 2007). Low levels of PA may worsen secondary disorders that are common in CP, such as musculoskeletal problems and poor cardiometabolic health (Peterson et al. 2013; Benner et al. 2019). As in the general population, PA at a young age is predictive of being physically active in adulthood in people with CP (Telama et al. 2005; Waltersson and Rodby‐Bousquet 2017). Therefore, supporting a physically active lifestyle early in life is essential.

Motor function varies greatly in people with CP, which, in turn, affects the level of PA. According to the Gross Motor Function Classification System (GMFCS), those with the mildest level of disability (GMFCS level I) walk without restrictions but have limitations in more advanced gross motor skills, whereas those with the most severe disability (GMFCS level V) use wheeled mobility and their self‐mobility is severely limited even with the use of assistive technology (Palisano et al. 1997). Several studies have shown that better gross motor functioning is associated with higher daily PA levels in all age groups in people with CP (Nieuwenhuijsen et al. 2009; T. A. Bania et al. 2014; Shkedy Rabani et al. 2014; Hulst et al. 2023). However, highly functioning and independently ambulatory people with GMFCS level I or II may be as active as their peers without mobility limitations (van den Berg‐Emons et al. 2010).

The current evidence about differences in PA levels across the gross motor function spectrum and between individuals with and without CP is, however, limited. The mechanical efficiency of, for example, walking, is lower in people with CP, leading to higher energy expenditure at given activity than in individuals without mobility limitations (van den Hecke et al. 2007; Nardon et al. 2021; Balemans et al. 2017). The energy expenditure is elevated in people with CP even if they have good gross motor function, and it is suggested to increase with increasing severity of motor function impairment (van den Hecke et al. 2007; Nardon et al. 2021; Johnston et al. 2004). The higher energy cost for a given motor task may be one explanation for lower activity levels in people with CP compared to those without physical disabilities (Balemans et al. 2017). Despite this, the differences in PA energy cost are overlooked in most studies (Aviram et al. 2019). Accelerometry is a useful tool to assess everyday PA, but the generally used raw accelerometry data processing algorithms do not account for individual differences in fitness and mobility (Strath et al. 2012). Therefore, GMFCS‐specific accelerometry data processing algorithms have been created for ambulatory people with CP (Trost et al. 2016).

Increasing moderate‐to‐vigorous PA even in short bouts is a key target in the current PA recommendations for individuals with CP (Verschuren et al. 2016). In addition, reducing sedentary time has been recognized as the most appropriate first‐line intervention preventing many of the secondary comorbidities in CP (Peterson et al. 2013). Physical training interventions are a promising method to increase daily PA in children and adolescents with CP (T. Bania et al. 2011; S. Reedman et al. 2017), although not all studies have found structured exercise programs effective (T. A. Bania et al. 2016; Ryan et al. 2020). However, research is lacking on the effects of exercise on time spent in sedentary, light, and moderate‐to‐vigorous PA in individuals with CP assessed with tailored GMFCS‐specific cutoffs.

Therefore, the purpose of the present study was to (1) investigate the volume of sedentary time, light PA, and moderate‐to‐vigorous PA among children and young adults with and without CP, while accounting for the lower movement efficiency associated with CP and (2) to investigate if PA changed during a 3‐month multicomponent exercise intervention among children and young adults with CP.

## 2 Materials and Methods

### 2.1 Study Design and Participants

This study reports secondary outcomes of the EXErcise for Cerebral Palsy (EXECP) study (Valadão et al. 2021, 2024), which used a nonconcurrent multiple baseline design. The multiple baseline design was selected instead of a randomized controlled trial design because the participants were expected to form a highly heterogeneous group and randomly dividing the sample into two groups would likely not have resulted in two comparable groups. In the multiple baseline design, the participants acted as their own controls (Valadão et al. 2021). The study protocol, recruitment of the participants, and results for the primary outcome, that is, the six‐minute walking distance, and other secondary outcomes, including measures of motor function, muscle strength, joint flexibility, and cardiometabolic risk factors, have been reported in detail in previous publications (Valadão et al. 2021, 2024; Savikangas et al. 2024).

Initially, 13 Finnish males and 7 females aged 9–22 with unilateral or bilateral spastic CP, classified as GMFCS levels I to III, were recruited to the CP group. Typically developing controls (TDs) were 17 age‐matched and sex‐matched children and young adults without any musculoskeletal disorders. The EXECP study included a 3‐month control period for both CP and TD groups. The CP group thereafter attended a 3‐month multicomponent exercise intervention, whereas TDs did not attend. Two participants in the CP group dropped out during the control period (Valadão et al. 2024).

The present substudy includes those participants who completed successfully accelerometer‐based PA assessment during the control period, that is, all participants with missing baseline PA data were excluded. The final analytical sample for research question 1 comprises seven male and three female participants with CP and eight TDs, six males and two females. Six participants with CP also completed the follow‐up accelerometry assessment and were included in the analysis for research question 2.

The EXECP study was registered prospectively in the International Standard Randomized Controlled Trial Registry (ISRCTN69044459), and the study protocol and the main results, that is, changes in six‐minute walking distance, have been published (Valadão et al. 2021, 2024). The study protocol was approved by the ethics committee of the Central Finland Healthcare District (U8/2017) and all participants and the legal guardians of the underaged participants signed informed consent before participation. Participants aged < 18 years gave written assent.

### 2.2 Physical Activity Assessment

PA was assessed with a triaxial accelerometer (models X6‐1a and X16‐1d, Gulf Coast Data Concepts Inc., Waveland, USA). The accelerometers stored acceleration data at 40–50 Hz with 12‐bit or 16‐bit resolution and an acceleration range of at least ± 2 g. Baseline assessments were performed during the 3‐month control period and follow‐up assessments during the last month of the 3‐month exercise intervention or immediately after the intervention. Participants received the accelerometers during the laboratory visits and were instructed to select a typical week for both the baseline and follow‐up assessments to minimize the potential impact from external factors (e.g., fatigue and schoolwork), ensuring a more accurate representation of habitual PA patterns. They were asked to wear the accelerometer for seven consecutive days during waking hours, except during water‐related activities (e.g., showering) in an elastic waistband above the iliac crest on the right side. Accelerometer use instructions were given both orally and in writing. When completing the follow‐up assessments during the intervention, participants did not wear accelerometers during the supervised training sessions.

Raw acceleration data were analyzed offline with custom‐written Python and MATLAB (version R2020b, The MathWorks Inc., Natick MA, USA) scripts. Raw data were analyzed for activity counts with an in‐house developed implementation of the Actigraphy count algorithms described by Neishabouri et al. (2022). The scripts are published and available online. Raw data were analyzed in fifteen‐second (15 s) nonoverlapping epochs for counts in accordance with previous studies (Trost et al. 2016; Gao et al. 2018). The epochs were divided into 24 h segments from midnight to midnight based on the time stamps.

For the 15 s epochs, the vector magnitude (VM) was analyzed as the square root of the sum of the squares of the three components. The VM count thresholds for sedentary time, light PA, and moderate‐to‐vigorous PA were chosen based on previous validation studies in adolescents with CP (Trost et al. 2016) and typically developing peers (Romanzini et al. 2014). Both the CP‐specific and general cutoffs have been defined and validated by measuring VO_2_ uptake and acceleration with the ActiGraph GT3X during different tasks (Trost et al. 2016; Romanzini et al. 2014). First, raw acceleration data analyses were performed for all participants both in the CP and in the TD groups with the general cutoffs (Romanzini et al. 2014). Second, the raw acceleration data of participants in the CP group were analyzed with tailored GMFCS‐specific cutoffs, which are suggested to discriminate light PA from sedentary behavior and moderate‐to‐vigorous PA from light PA more accurately in young people with CP compared to the general cutoffs (Trost et al. 2016). The general and GMFCS‐specific VM count thresholds used to define sedentary time, light PA, and moderate‐to‐vigorous PA are summarized in Table 1. Nonwear time was taken off as any epoch of at least 60 min with the 15 s count values continuously being zero.

**TABLE 1:** General and GMFCS‐specific vector magnitude count thresholds for sedentary, light, and moderate‐to‐vigorous intensity physical activity.

	Sedentary time	Light PA	Moderate‐to‐vigorous PA
General (Romanzini et al. 2014)	0–180	> 180–756	> 756
GMFCS‐specific (Trost et al. 2016)			
GMFCS I	0–72	> 72–724	> 724
GMFCS III	0–72	> 72–669	> 669

Abbreviations: GMFCS = Gross Motor Function Classification System, PA = physical activity.

The 15 s epochs were used to calculate the daily volumes of sedentary time, light PA, and moderate‐to‐vigorous intensity PA. After that, average daily minutes in intensity categories were calculated from the days considered acceptable, that is, days with at least 8 h of wear time (Cain et al. 2013). At least three acceptable days were required for the assessment to be included in the analysis. Finally, the average daily proportions of wear time spent in sedentary time, light PA, and moderate‐to‐vigorous PA were calculated.

We excluded all participants, who did not successfully complete the baseline PA assessment (i.e., ≥ 3 days with ≥ 8 h of wear time) during the control period. Six of the 10 participants with CP who had valid baseline data also completed the follow‐up assessment successfully. Participant noncompliance and device malfunction, caused by the operator (i.e., the participant thought the device was active, but it was not) and by the device (e.g., file corruption and incorrect acquisition settings), were the main reasons for the high amount of missing data.

### 2.3 Descriptive Characteristics

Sex and date of birth were self‐reported. Weight (kg) was assessed with a digital scale and height (cm) with a wall‐mounted stadiometer. Walking ability was assessed with the distance completed in the six‐minute walking test.

### 2.4 Statistical Methods

Participant characteristics in each group are summarized as means and standard deviations (SDs) for continuous variables and frequencies (*N*) and percentages for categorical variables. Between‐group differences in participant characteristics were tested with independent samples two‐tailed *t*‐tests for continuous variables and chi‐squared tests for categorical variables. Although not all participants in the CP and TD groups included in the present study were matched pairs according to the original sampling design, preliminary analyses suggested that mean age and sex distribution were comparable in the study groups. Therefore, there was no need to adjust further statistical analyses for age or sex.

Differences in PA outcomes between TDs and participants with CP were investigated with independent samples *t*‐tests. Within the CP group, differences in the volume of PA in each intensity category according to the general and GMFCS cutoffs were compared with paired samples *t*‐tests. Paired samples *t*‐tests were also conducted to investigate changes in PA from baseline to follow‐up in participants with CP. The results are presented as means and SDs or 95% confidence intervals (CIs). Cohen's *d* was calculated to estimate the effect size (ES). All statistical analyses were performed with the jamovi software 2.2.5 (jamovi). The power calculations were based on the main outcome of the EXECP study, that is, the six‐minute walking distance, and were not extended to the secondary outcomes, including measures of PA (Valadão et al. 2021).

## 3 Results

Participant characteristics at baseline are summarized in Table 2 using the study group. There were no statistically significant between‐group differences in age, anthropometric measures, or sex, whereas TDs had a significantly longer six‐minute walking distance compared to participants with CP.

**TABLE 2:** Participant characteristics.

	TD	CP	*p*
	Mean ± SD	Mean ± SD	
Age, years	15.3 ± 4.2	15.4 ± 5.1	0.968
Weight, kg	57.5 ± 19.5	50.4 ± 13.7	0.374
Height, cm	164.2 ± 19.2	158.9 ± 14.1	0.513
BMI, kg/m^2^	20.8 ± 4.0	19.6 ± 2.9	0.456
Six‐minute walking distance, m	735.8 ± 129.2	488.6 ± 140.7	0.001

	*N* (%)	*N* (%)	
Sex			
Male	6 (75)	7 (70)	0.814
Female	2 (25)	3 (30)	
GMFCS			
I	—	7 (70)	
III	—	3 (30)	
Type of CP			—
Unilateral	—	5 (50)	
Bilateral	—	5 (50)	

Abbreviations: BMI = body mass index, CP = cerebral palsy, GMFCS = Gross Motor Function Classification System, SD = standard deviation, TD = typically developing.

PA minutes and proportions of time spent in each intensity category of the overall accelerometer wear time are presented in Table 3. Average daily wear time was 12.4 (SD 2.0) hours in the TDs and 13.4 (1.0) hours per day in the participants with CP, but the difference was not statistically significant (*p* = 0.171). When applying the general PA intensity cutoffs to all participants, mean sedentary time was on average 8.6 and 10.2 h per day in the TDs and participants with CP, respectively. Despite the large effect size (Cohen's *d* = −0.88), the 95% CI of the 1.5 h difference exceeded zero. The difference in sedentary time between the groups attenuated when comparing the proportion of sedentary time of the wear time: TDs spent on average 69% of the wear time in sedentary activities, whereas the corresponding proportion for the participants with CP was 76%.

**TABLE 3:** Physical activity at baseline in the participants with cerebral palsy (CP) and typically developing (TD) controls.

	TD_GEN_	CP_GEN_	TD_GEN_–CP_GEN_			CP_GMFCS_	TD_GEN_–CP_GMFCS_			CP_GEN_–CP_GMFCS_		
	Mean ± SD	Mean ± SD	Mean difference [95% CI]	ES	*p*	Mean ± SD	Mean difference [95% CI]	ES	*p*	Mean difference [95% CI]	ES	*p*
**Min/d**					0.171							
Sedentary time	516.0 ± 122.1	610.0 ± 92.3	−94.0 [−201.0, 12.9}	−0.88	0.081	537.2 ± 105.4	−21.2 [−134.8, 92.4]	−0.19	0.698	72.83 [60.21, 85.45]	4.13	< 0.001
Light PA	154.5 ± 37.3	138.4 ± 46.3	16.2 [−26.7, 59.0]	0.38	0.436	205.6 ± 60.7	−51.1 [−103.2, 1.0]	−0.99	0.054	−67.27 [−79.68, −54.87}	−3.88	< 0.001
Moderate‐to‐vigorous PA	75.5 ± 28.3	58.0 ± 29.5	17.6 [−11.6, 46.7]	0.61	0.220	63.5 ± 30.8	12.0 [−17.9, 41.9]	0.40	0.408	−5.56 [−8.45, −2.66]	−1.37	0.002
**% Of wear time**												
Sedentary time	68.7 ± 8.8	75.5 ± 8.8	−6.8 [−15.6, 2.0]	−0.77	0.631	66.4 ± 10.7	2.3 [−7.6, 12.2]	0.23	0.631	9.09 [7.45, 10.73}	3.97	< 0.001
Light PA	20.8 ± 5.0	17.2 ± 5.7	3.6 [−1.8, 9.2]	0.67	0.143	25.6 ± 7.4	−4.8 [−11.3, 1.8]	−0.73	0.143	−8.39 [−9.94, −6.84}	−3.88	< 0.001
Moderate‐to‐vigorous PA	10.4 ± 4.5	7.3 ± 3.8	3.2 [−1.0, 7.3]	0.77	0.237	8.0 ± 4.0	2.5 [−1.8, 6.7]	0.58	0.237	−0.70 [−1.11, −0.30]	−1.23	0.004

Abbreviations: CI = confidence interval, CP_GEN_ = general physical activity intensity category thresholds applied to participants with CP, CP_GMFCS_ = Gross Motor Function Classification System (GMFCS)—specific physical activity intensity thresholds applied to participants with CP, ES = Cohen's *d* effect size, PA = physical activity, SD = standard deviation, TD_GEN_ = general physical activity intensity category thresholds applied to typically developing participants.

The TDs had on average 2.6 and 1.2 h of light and moderate‐to‐vigorous PA per day, corresponding to 21% and 10% of the wear time, respectively. When applying the general PA intensity cutoffs to the participants with CP, they accumulated an average of 2.3 and 1.0 h of light and moderate‐to‐vigorous PA per day, that is, 17% and 7% of the daily wear time, respectively. Light or moderate‐to‐vigorous PA did not differ between the groups when applying the general intensity cutoffs to all participants.

When the GMFCS‐specific cutoffs for light and moderate‐to‐vigorous PA were applied to the participants with CP, they recorded on average 9.0, 3.4, and 1.0 h of sedentary time, light PA, and moderate‐to‐vigorous PA per day, respectively. They had on average 51 min more of light PA per day than the TDs (TD‐CP = −51 min, Cohen's *d* = −0.99, and 95% CI [−103.2, 1.0]). Daily minutes of sedentary time or moderate‐to‐vigorous PA or the proportions of sedentary time, light PA, or moderate‐to‐vigorous PA of the daily wear time did not differ between the TDs and participants with CP when applying the GMFCS‐specific cutoffs.

Comparing the daily volume of PA in intensity categories within the CP group based on the general and GMFCS‐specific cutoffs revealed statistically significant differences in all outcomes. Using the GMFCS‐specific cutoffs yielded lower levels of sedentary time and higher amounts of light and moderate‐to‐vigorous PA as both total minutes per day and the proportion of wear time (*p* ≤ 0.004 for all; Table 3).

In participants with CP, the average daily wear time, total daily volume of sedentary time, light PA, moderate‐to‐vigorous PA, or the proportions of PA in intensity categories of the daily wear time did not change from baseline to follow‐up (Table 4).

**TABLE 4:** Changes in physical activity from baseline to follow‐up in participants with CP based on GMFCS‐specific cutoffs.

	Baseline	Follow‐up	Change^a^		
	Mean ± SD	Mean ± SD	Mean difference [95% CI]	ES	*p*
Wear time, h	13.3 ± 1.2	12.6 ± 1.3	−0.73 [−2.5, 1.0]	−0.44	0.327
Sedentary time, min	561.1 ± 119.4	528.5 ± 115.4	−32.5 [−132.5, 67.4)	−0.34	0.441
Light PA, min	183.7 ± 62.5	169.7 ± 64.7	−14.1 [−31.7, 3.7]	−0.83	0.098
Moderate‐to‐vigorous PA, min	53.3 ± 29.9	55.8 ± 30.7	2.5 [−15.1, 20.2]	0.15	0.727
Sedentary time, % of wear time	70.0 ± 11.3	69.9 ± 12.9	−0.1 [6.2,6.0]	−0.02	0.962
Light PA, % of wear time	23.2 ± 7.8	22.8 ± 9.9	−0.4 [−5.3, 4.4]	−0.09	0.839
Moderate‐to‐vigorous PA, % of wear time	6.8 ± 4.2	7.3 ± 3.8	0.5 [−1.8, 2.8]	0.23	0.590

Abbreviations: CI = confidence interval, ES = Cohen's *d* effect size, PA = physical activity, SD = standard deviation.

^a^ Change calculated as follow‐up—baseline.

## 4 Discussion

In the present study, children and young adults with CP achieved the recommended amount of 1 h of moderate‐to‐vigorous PA per day. When accounting for the daily accelerometer wear time, the proportions of sedentary time, light PA, and moderate‐to‐vigorous PA did not differ from their typically developing peers, independent of the choice of PA intensity category cutoffs applied to the raw acceleration data. On the contrary, the choice of the cutoff affected the results, when investigating the differences in the absolute daily volumes of sedentary time and light PA. Although participants with CP accumulated on average more sedentary time than typically developing controls when the general cutoffs were applied to all participants, they had more light PA, when their higher PA energy expenditure was considered, and the GMFCS‐specific cutoffs were used. In the participants with CP, the distribution of daily PA into sedentary time, light PA, and moderate‐to‐vigorous PA did not change during the 3‐month exercise intervention.

Previous research has shown that energy expenditure during a given activity, such as walking, is higher in people with CP compared to TDs (Nardon et al. 2021). Despite this, only a few studies have investigated the PA levels of children and young adults with CP with accelerometry data processing approaches that consider their increased energy cost. When applying previously validated GMFCS‐specific cutoffs to the participants with CP in the present study (Trost et al. 2016), their mean daily time spent sedentary, and in light and moderate‐to‐vigorous PA, was comparable to previous studies, which utilized the same cutoff values (Ryan et al. 2020; S. E. Reedman et al. 2019; Lavelle et al. 2020; Yoon et al. 2022). It is noteworthy that the choice of PA intensity category cutoffs affected notably the amount of sedentary time and light PA in participants with CP. Applying the GMFCS‐specific cutoffs yielded approximately 73 min less sedentary time, 67 min more light PA, and 6 min more moderate‐to‐vigorous PA per day compared to the general cutoffs previously validated in typically developing adolescents (Romanzini et al. 2014). This finding is of importance since all daily activity is considered beneficial for children and young adults with CP (Verschuren et al. 2016), and appropriate PA assessment methods are necessary to accurately assess activity levels and the effectiveness of PA promotion interventions in individuals with mobility limitations both in research and clinical practice.

The choice of intensity cutoffs for participants with CP affected the results considering differences in PA between the groups. When applying the general cutoffs to all participants, we did not find differences in light or moderate‐to‐vigorous PA between the groups, which is in contrast to most (Capio et al. 2012; Obeid et al. 2014; Okur et al. 2021; Nooijen et al. 2014; Aviram et al. 2019; Nieuwenhuijsen et al. 2009) but not all (Salie et al. 2021) previous studies. However, the participants with CP accumulated more sedentary time per day than TDs, which was mostly explained by their longer average daily accelerometer wear time. This is supported by the finding that the difference in sedentary time diminished when inspected in relation to wear time. The 1.0 and 1.5 h differences in mean daily wear time and sedentary time, respectively, between the groups were relatively large (effect sizes −0.7 and −0.9, respectively) but not statistically significant due to the small sample size and high variation in these outcomes.

The results changed when applying the GMFCS‐specific raw acceleration data processing approach with a lower cutoff for distinguishing light PA from sedentary time for participants with CP: they then accumulated approximately 50 min more of light PA per day compared to TDs, although the between‐group difference was not significant when exploring the proportion of light PA of the total wear time. This finding is likely explained by the longer wear time emphasizing the proportion of sedentary time over active time in the participants with CP compared to TDs. It is noteworthy that there were no statistically significant differences in moderate‐to‐vigorous PA independent of the selected cutoffs. The small sample size and high variability in the outcomes may partly explain the nonsignificant findings despite moderate‐to‐large effect sizes.

To our knowledge, there are no previous studies that compare the level of PA between participants with and without CP by applying intensity cutoffs based on motor function and these results provide novel insights to the importance of choices made in accelerometry data processing in individuals with motor disabilities. Although the use of GMFCS‐specific intensity cutoffs is justified due to the increased energy expenditure of, for example, equally paced walking, in people with CP (Nardon et al. 2021), it would be important to conduct a validation study involving children and young adults with and without CP in the same procedure to investigate, if the difference in energy expenditure of light‐intensity activities, such as standing household chores, is as large as indicated by the thresholds of 72 and 180 VM counts per 15 s, that are suggested to distinguish light PA from sedentary time in adolescents with and without CP, respectively (Trost et al. 2016; Romanzini et al. 2014). The cutoffs applied to TDs were chosen because they had been validated in a comparable group of adolescents with the same accelerometer model as the cutoffs for adolescents with CP. However, the activity protocols of the validation studies differed for the amount and type of activities included. The one used to validate cutoffs in a general adolescent population included walking and running at predefined paces and sporting activities (e.g., soccer) and a greater number of different activities overall (Romanzini et al. 2014). In contrast, the activity protocol for adolescents with CP included household tasks and walking activities mimicking different situations at different self‐selected paces (Trost et al. 2016). These differences make the cutoffs not fully comparable and may, to some extent, explain the results.

Another explanation for why our findings deviate from the main body of existing literature suggesting lower PA and higher sedentary time in children and young adults with CP compared to TDs may lie in selection bias. The children and young adults with CP in the present study had agreed to participate in an intensive 3‐month exercise intervention and were relatively active, accumulating approximately 1 h of moderate‐to‐vigorous‐ PA per day, already at baseline. It is thus possible that they were more physically active than children and young adults with CP in general. However, it is to be noted that in comparison to a general population of Finnish children and adolescents of the age 9–15 years, who accumulate on average 3.5 h of light and 1.5 h of moderate‐to‐vigorous activity per day (Jussila et al. 2022), the mean daily PA of the participants with CP in the present study was lower. This supports the findings of another Finnish population‐based study, which showed that adolescents with disabilities were slightly less active than those without disabilities (Ng et al. 2019). The present study extends the results of the study of Ng and colleagues (Ng et al. 2019) by focusing on PA in Finnish children and young adults with CP, whose PA levels have not been studied previously.

In our previous study, we found that the 3‐month exercise intervention focusing on strength training and improving gait quality, increased motor function, strength, and flexibility in the participants with CP (Valadão et al. 2024). However, the present study suggests that the intervention was not successful in increasing daily PA. The existing evidence about the impact of exercise interventions on daily PA in children and young adults with CP is mixed. Our findings are in line with a few previous studies investigating the effects of diverse exercise interventions on PA in 8–19‐year‐old children and young adults, which did not increase their daily step counts, light PA, moderate‐to‐vigorous PA, or reduce the sedentary time (T. A. Bania et al. 2016; Ryan et al. 2020; S. E. Reedman et al. 2019; Mitchell et al. 2016). However, in other studies, treadmill gait training and strength training have been successful in increasing the daily step counts in 6–20‐year‐old children and young adults (Bjornson et al. 2019; Bar‐Haim et al. 2019). There are a few plausible explanations for why the intervention did not increase PA and reduce sedentary time. The participants with CP were already relatively active at the baseline, and there may thus not have been much room for improvement in habitual activity while increasing the amount of supervised exercising, which was not included in the follow‐up accelerometry recordings. It is also important to consider the nature of the intervention. The EXECP intervention focused on strength, flexibility, and gait training, which may not directly translate into increased spontaneous daily‐life PA, as this is related to personal PA habits. It should be acknowledged that exercise training takes only a fraction of the day and has limited capacity to change the PA profile of the entire day (Finni et al. 2016). Furthermore, the loss of data were very large when investigating the effects of the intervention on PA: only six of the 18 participants who completed the intervention had valid accelerometry data from both baseline and follow‐up measurements. The null findings may thus partly be explained by a lack of statistical power, which is also reflected in the fact that some effect sizes for change in daily PA minutes were moderate or large, such as Cohen's *d* −0.83, indicating a decline in light PA. However, although wear time showed a nonsignificant decrease from baseline to follow‐up, the effect sizes for change in the proportional distribution of sedentary and active time showed only small effect sizes, indicating that the intervention was not effective. Future research should explore whether complementary interventions—such as physical activity promotion strategies alongside structured exercise training—are needed to drive meaningful increases in PA in this population.

This study has several limitations. Due to noncompliance and technical issues, the amount of missing data were large. Participants were instructed to use the accelerometers during a typical week both during the control period and follow‐up to minimize fluctuations due to external factors. However, this led to participant noncompliance, technical failures, and variability in the timing of the accelerometry. Due to the lack of a within‐subject control period for physical activity and a nonexercising control group, we cannot draw solid conclusions on the impact of the intervention on PA. Since the power calculations were based on the main outcome of the EXECP study, that is, the six‐minute walking distance, and the final sample size of the present study was small, the analyses lacked statistical power. Furthermore, due to the small sample size, we could not investigate PA levels in subgroups, such as using sex, age, GMFCS level, or type of CP. To reach a sufficient number of participants in the parent trial, the age range had to be wide, ranging from children to young adults. As it is generally known that PA typically declines from childhood to adulthood (Corder et al. 2019; van Sluijs et al. 2021), this may have increased the within‐group variability. There was some variation in accelerometer models and their calibration during a long data collection period, but based on inspection of raw accelerometry data, this did not cause any problems. It must also be noted that the results from both the within‐group and between‐group analyses must be interpreted with some caution due to a large amount of missing data, potentially resulting in nonrepresentative samples, and the differences in accelerometer wear compliance between the groups and, in the CP group, the measurements, which may affect especially the proportional PA measures. Finally, the participants with CP and their families were motivated to participate in an intensive exercise intervention and were probably more active than individuals with CP in general, which limits the generalizability of the results.

Despite these limitations, this study also has strengths. We applied two different sets of cutoffs to investigate the level of PA in individuals with CP and to compare their PA levels to TDs. This approach revealed the importance of the choice of PA intensity cutoffs in this population and suggests that in the future, it would be important to use equal validation processes to determine intensity cutoffs in individuals with CP and TDs to allow more accurate comparison of PA levels in these groups. This study also provides novel information about PA in Finnish children and young adults with CP. Despite CP being the most common permanent disability diagnosed in childhood also in Finland, PA levels have not been previously studied in this population.

## 5 Conclusion

This study showed that it is important to consider the higher physical activity energy cost of individuals with CP. Children and young adults with CP may be as active as their typically developing peers, especially when their higher physical activity energy cost is accounted for. Muscle strength and gait performance improving exercise did not seem promising in facilitating habitual physical activity. Other types of physical activity promotion programs are needed to increase daily physical activity in children and young adults with CP.

## Author Contributions

**Tiina Savikangas:** conceptualization, data curation, formal analysis, methodology, writing – original draft. **Pedro Valadão:** conceptualization, data curation, funding acquisition, investigation, methodology, project administration, writing – review and editing. **Eero A. Haapala:** conceptualization, data curation, investigation, methodology, writing – review and editing. **Iida Laatikainen‐Raussi:** data curation, formal analysis, methodology, writing – review and editing. **Timo Rantalainen:** data curation, methodology, writing – review and editing. **Taija Finni:** conceptualization, data curation, funding acquisition, investigation, methodology, project administration, supervision, writing – review and editing.

## Ethics Statement

The EXECP study protocol received a positive ethical statement from the ethics committee of the Central Finland Healthcare District (U8/2017).

## Consent

All participants and the legal guardians of the underaged participants signed a written informed consent.

## Conflicts of Interest

The authors declare no conflicts of interest.

## Data Availability

Data and the analysis outputs will be made available in the Open Science Framework repository (DOI 10.17605/OSF.IO/4KBJH). The raw accelerometry data processing scripts are published and available online (https://gitlab.jyu.fi/tjrantal/jyu_actigraphy_analysis).

## References

[ejsc12313-bib-0001] Aviram, R. , N. Harries , A. S. Rabani , et al. 2019. “Comparison of Habitual Physical Activity and Sedentary Behavior in Adolescents and Young Adults With and Without Cerebral Palsy.” Pediatric Exercise Science 31, no. 1: 60–66. 10.1123/pes.2017-0285. 30272530

[ejsc12313-bib-0002] Balemans, A. C. , E. A. Bolster , M.‐A. Brehm , and A. J. Dallmeijer . 2017. “Physical Strain: A New Perspective on Walking in Cerebral Palsy.” Archives of Physical Medicine and Rehabilitation 98, no. 12: 2507–2513. 10.1016/j.apmr.2017.05.004. 28596080

[ejsc12313-bib-0003] Bania, T. , K. J. Dodd , and N. Taylor . 2011. “Habitual Physical Activity Can Be Increased in People With Cerebral Palsy: A Systematic Review.” Clinical Rehabilitation 25, no. 4: 303–315. 10.1177/0269215510383062. 21078699

[ejsc12313-bib-0004] Bania, T. A. , K. J. Dodd , R. J. Baker , H. K. Graham , and N. F. Taylor . 2016. “The Effects of Progressive Resistance Training on Daily Physical Activity in Young People With Cerebral Palsy: A Randomised Controlled Trial.” Disability & Rehabilitation 38, no. 7: 620–626. 10.3109/09638288.2015.1055376. 26056856

[ejsc12313-bib-0005] Bania, T. A. , N. F. Taylor , R. J. Baker , H. K. Graham , L. Karimi , and K. J. Dodd . 2014. “Gross Motor Function Is an Important Predictor of Daily Physical Activity in Young People With Bilateral Spastic Cerebral Palsy.” Developmental Medicine and Child Neurology 56, no. 12: 1163–1171. 10.1111/dmcn.12548. 25052563

[ejsc12313-bib-0006] Bar‐Haim, S. , R. Aviram , A. S. Rabani , et al. 2019. “Effects of Exercise Interventions on Habitual Physical Activity and Sedentary Behavior in Adolescents With Cerebral Palsy.” Pediatric Exercise Science 31, no. 4: 416–424. 10.1123/pes.2018-0254. 30922152

[ejsc12313-bib-0007] Benner, J. L. , P. G. McPhee , J. W. Gorter , et al. 2019. “Focus on Risk Factors for Cardiometabolic Disease in Cerebral Palsy: Toward a Core Set of Outcome Measurement Instruments.” Archives of Physical Medicine and Rehabilitation 100, no. 12: 2389–2398. 10.1016/j.apmr.2019.04.012. 31128113

[ejsc12313-bib-0008] Bjornson, K. F. , N. Moreau , and A. W. Bodkin . 2019. “Short‐Burst Interval Treadmill Training Walking Capacity and Performance in Cerebral Palsy: A Pilot Study.” Developmental Neurorehabilitation 22, no. 2: 126–133. 10.1080/17518423.2018.1462270. 29658831 PMC7894036

[ejsc12313-bib-0009] Bull, F. C. , S. S. Al‐Ansari , S. Biddle , et al. 2020. “World Health Organization 2020 Guidelines on Physical Activity and Sedentary Behaviour.” British Journal of Sports Medicine 54, no. 24: 1451–1462. 10.1136/bjsports-2020-102955. 33239350 PMC7719906

[ejsc12313-bib-0010] Cain, K. L. , J. F. Sallis , T. L. Conway , D. Van Dyck , and L. Calhoon . 2013. “Using Accelerometers in Youth Physical Activity Studies: A Review of Methods.” Journal of Physical Activity and Health 10, no. 3: 437–450. 10.1123/jpah.10.3.437. 23620392 PMC6331211

[ejsc12313-bib-0011] Capio, C. M. , C. H. P. Sit , B. Abernethy , and R. S. W. Masters . 2012. “Fundamental Movement Skills and Physical Activity Among Children With and Without Cerebral Palsy.” Research in Developmental Disabilities 33, no. 4: 1235–1241. 10.1016/j.ridd.2012.02.020. 22502850

[ejsc12313-bib-0012] Chaput, J.‐P. , J. Willumsen , F. Bull , et al. 2020. “2020 WHO Guidelines on Physical Activity and Sedentary Behaviour for Children and Adolescents Aged 5–17 Years: Summary of the Evidence.” International Journal of Behavioral Nutrition and Physical Activity 17, no. 1: 141. 10.1186/s12966-020-01037-z. 33239009 PMC7691077

[ejsc12313-bib-0013] Corder, K. , E. Winpenny , R. Love , H. E. Brown , M. White , and E. van Sluijs . 2019. “Change in Physical Activity From Adolescence to Early Adulthood: A Systematic Review and Meta‐Analysis of Longitudinal Cohort Studies.” British Journal of Sports Medicine 53, no. 8: 496–503. 10.1136/bjsports-2016-097330. 28739834 PMC6250429

[ejsc12313-bib-0014] Finni, T. , M. Uusi‐Vähälä , A. J. Pesola , and R. S. Taipale . 2016. “Do Running and Strength Exercises Reduce Daily Muscle Inactivity Time?” AIMS Public Health 3, no. 4: 702–721. 10.3934/publichealth.2016.4.702. 29546190 PMC5690400

[ejsc12313-bib-0015] Gao, Y. , M. Melin , K. Mäkäräinen , et al. 2018. “Children's Physical Activity and Sedentary Time Compared Using Assessments of Accelerometry Counts and Muscle Activity Level.” PeerJ 6: e5437. 10.7717/peerj.5437. 30155355 PMC6108314

[ejsc12313-bib-0016] Guthold, R. , G. A. Stevens , L. M. Riley , and F. C. Bull . 2018. “Worldwide Trends in Insufficient Physical Activity From 2001 to 2016: A Pooled Analysis of 358 Population‐Based Surveys With 1· 9 Million Participants.” Lancet Global Health 6, no. 10: e1077–e1086. 10.1016/s2214-109x(18)30357-7. 30193830

[ejsc12313-bib-0017] Guthold, R. , G. A. Stevens , L. M. Riley , and F. C. Bull . 2020. “Global Trends in Insufficient Physical Activity Among Adolescents: A Pooled Analysis of 298 Population‐Based Surveys With 1·6 Million Participants.” Lancet Child & Adolescent Health 4, no. 1: 23–35. 10.1016/S2352-4642(19)30323-2. 31761562 PMC6919336

[ejsc12313-bib-0018] Hulst, R. Y. , J. W. Gorter , J. Obeid , et al. 2023. “Accelerometer‐measured Physical Activity, Sedentary Behavior, and Sleep in Children With Cerebral Palsy and Their Adherence to the 24‐hour Activity Guidelines.” Developmental Medicine and Child Neurology 65, no. 3: 393–405. 10.1111/dmcn.15338. 35833425 PMC10084309

[ejsc12313-bib-0019] “Jamovi ‐ Open Statistical Software for the Desktop and Cloud.” Accessed January 26, 2024. https://www.jamovi.org/.

[ejsc12313-bib-0020] Johnston, T. E. , S. E. Moore , L. T. Quinn , and B. T. Smith . 2004. “Energy Cost of Walking in Children With Cerebral Palsy: Relation to the Gross Motor Function Classification System.” Developmental Medicine and Child Neurology 46, no. 1: 34–38. 10.1017/s0012162204000064. 14974645

[ejsc12313-bib-0021] Jussila, A.‐M. , P. Husu , H. Vähä‐Ypyä , et al. 2022. “Accelerometer‐Measured Physical Activity Levels and Patterns Vary in an Age‐ and Sex‐Dependent Fashion Among Finnish Children and Adolescents.” International Journal of Environmental Research and Public Health 19, no. 11: 6950. 10.3390/ijerph19116950. 35682533 PMC9180141

[ejsc12313-bib-0022] Lavelle, G. , M. Noorkoiv , N. Theis , et al. 2020. “Validity of the International Physical Activity Questionnaire Short Form (IPAQ‐SF) as a Measure of Physical Activity (PA) in Young People With Cerebral Palsy: A Cross‐Sectional Study.” Physiotherapy 107: 209–215. 10.1016/j.physio.2019.08.013. 32026822

[ejsc12313-bib-0023] Mitchell, L. E. , J. Ziviani , and R. N. Boyd . 2016. “A Randomized Controlled Trial of Web‐Based Training to Increase Activity in Children With Cerebral Palsy.” Developmental Medicine and Child Neurology 58, no. 7: 767–773. 10.1111/dmcn.13065. 26877078

[ejsc12313-bib-0024] Nardon, M. , F. Ruzzante , L. O'Donnell , A. Adami , S. Dayanidhi , and M. Bertucco . 2021. “Energetics of Walking in Individuals With Cerebral Palsy and Typical Development, Across Severity and Age: A Systematic Review and Meta‐Analysis.” Gait & Posture 90: 388–407. 10.1016/j.gaitpost.2021.09.190. 34564011

[ejsc12313-bib-0025] Neishabouri, A. , J. Nguyen , J. Samuelsson , et al. 2022. “Quantification of Acceleration as Activity Counts in ActiGraph Wearable.” Scientific Reports 12, no. 1: 11958. 10.1038/s41598-022-16003-x. 35831446 PMC9279376

[ejsc12313-bib-0026] Ng, K. W. , P. Rintala , P. Husu , J. Villberg , T. Vasankari , and S. Kokko . 2019. “Device‐Based Physical Activity Levels Among Finnish Adolescents With Functional Limitations.” Disability and Health Journal 12, no. 1: 114–120. 10.1016/j.dhjo.2018.08.011. 30243486

[ejsc12313-bib-0027] Nieuwenhuijsen, C. , W. M. A. van der Slot , A. Beelen , et al. 2009. “Inactive Lifestyle in Adults With Bilateral Spastic Cerebral Palsy.” Journal of Rehabilitation Medicine 41, no. 5: 375–381. 10.2340/16501977-0340. 19363572

[ejsc12313-bib-0028] Nooijen, C. , J. Slaman , W. Slot , H. Stam , M. Roebroeck , and R. van den Berg‐Emons . 2014. “Health‐Related Physical Fitness of Ambulatory Adolescents and Young Adults With Spastic Cerebral Palsy.” Journal of Rehabilitation Medicine 46, no. 7: 642–647. 10.2340/16501977-1821. 24714702

[ejsc12313-bib-0029] Obeid, J. , A. C. J. Balemans , S. G. Noorduyn , J. W. Gorter , and B. W. Timmons . 2014. “Objectively Measured Sedentary Time in Youth With Cerebral Palsy Compared With Age‐Sex‐And Season‐Matched Youth Who Are Developing Typically: An Explorative Study.” Physical Therapy 94, no. 8: 1163–1167. 10.2522/ptj.20130333. 24652472

[ejsc12313-bib-0030] Okur, E. O. , D. Inal‐Ince , M. Saglam , N. Vardar‐Yagli , and H. Arikan . 2021. “Physical Activity Patterns in Children With Cerebral Palsy and Typically Developing Peers.” Physiotherapy Theory and Practice 37, no. 6: 710–718. 10.1080/09593985.2019.1641863. 31298601

[ejsc12313-bib-0031] Palisano, R. , P. Rosenbaum , S. Walter , D. Russell , E. Wood , and B. Galuppi . 1997. “Development and Reliability of a System to Classify Gross Motor Function in Children With Cerebral Palsy.” Developmental Medicine and Child Neurology 39, no. 4: 214–223. 10.1111/j.1469-8749.1997.tb07414.x. 9183258

[ejsc12313-bib-0032] Peterson, M. D. , P. M. Gordon , and E. A. Hurvitz . 2013. “Chronic Disease Risk Among Adults With Cerebral Palsy: The Role of Premature Sarcopoenia, Obesity and Sedentary Behaviour.” Obesity Reviews 14, no. 2: 171–182. 10.1111/j.1467-789X.2012.01052.x. 23094988

[ejsc12313-bib-0033] Reedman, S. , R. N. Boyd , and L. Sakzewski . 2017. “The Efficacy of Interventions to Increase Physical Activity Participation of Children With Cerebral Palsy: A Systematic Review and Meta‐Analysis.” Developmental Medicine and Child Neurology 59, no. 10: 1011–1018. 10.1111/dmcn.13413. 28318009

[ejsc12313-bib-0034] Reedman, S. E. , R. N. Boyd , S. G. Trost , C. Elliott , and L. Sakzewski . 2019. “Efficacy of Participation‐Focused Therapy on Performance of Physical Activity Participation Goals and Habitual Physical Activity in Children With Cerebral Palsy: A Randomized Controlled Trial.” Archives of Physical Medicine and Rehabilitation 100, no. 4: 676–686. 10.1016/j.apmr.2018.11.012. 30543803

[ejsc12313-bib-0035] Romanzini, M. , E. L. Petroski , D. Ohara , A. C. Dourado , and F. F. Reichert . 2014. “Calibration of ActiGraph GT3X, Actical and RT3 Accelerometers in Adolescents.” European Journal of Sport Science 14, no. 1: 91–99. 10.1080/17461391.2012.732614. 24533499

[ejsc12313-bib-0036] Rosenbaum, P. , N. Paneth , A. Leviton , et al. 2007. “A Report: The Definition and Classification of Cerebral Palsy April 2006.” Developmental Medicine & Child Neurology ‐ Supplement 109: 8–14. 17370477

[ejsc12313-bib-0037] Ryan, J. M. , G. Lavelle , N. Theis , et al. 2020. “Progressive Resistance Training for Adolescents With Cerebral Palsy: The STAR Randomized Controlled Trial.” Developmental Medicine and Child Neurology 62, no. 11: 1283–1293. 10.1111/dmcn.14601. 32588919

[ejsc12313-bib-0038] Salie, R. , M. M. Eken , K. A. Donald , A. G. Fieggen , and N. G. Langerak . 2021. “Physical Activity Levels of Adolescents and Adults With Cerebral Palsy in Urban South Africa.” Frontiers in Neurology 12: 747361. 10.3389/fneur.2021.747361. 34777217 PMC8581637

[ejsc12313-bib-0039] Savikangas, T. , P. Valadão , E. A. Haapala , and T. Finni . 2024. “Effects of Multicomponent Exercise Intervention on Cardiometabolic Risk Factors in Children and Young Adults With Cerebral Palsy: A Multiple‐Baseline Trial.” BMC Sports Science, Medicine and Rehabilitation 16, no. 1: 219. 10.1186/s13102-024-01006-0. PMC11492564 39434176

[ejsc12313-bib-0040] Shkedy Rabani, A. , N. Harries , I. Namoora , M. D. Al‐Jarrah , A. Karniel , and S. Bar‐Haim . 2014. “Duration and Patterns of Habitual Physical Activity in Adolescents and Young Adults With Cerebral Palsy.” Developmental Medicine and Child Neurology 56, no. 7: 673–680. 10.1111/dmcn.12394. 24506509

[ejsc12313-bib-0041] Strath, S. J. , K. A. Pfeiffer , and M. C. Whitt‐Glover . 2012. “Accelerometer Use With Children, Older Adults, and Adults With Functional Limitations.” Supplement, Medicine & Science in Sports & Exercise 44, no. 1 Suppl 1: S77–S85. 10.1249/MSS.0b013e3182399eb1. 22157778 PMC3292184

[ejsc12313-bib-0042] Telama, R. , X. Yang , J. Viikari , I. Välimäki , O. Wanne , and O. Raitakari . 2005. “Physical Activity From Childhood to Adulthood: A 21‐Year Tracking Study.” American Journal of Preventive Medicine 28, no. 3: 267–273. 10.1016/j.amepre.2004.12.003. 15766614

[ejsc12313-bib-0043] Trost, S. G. , M. Fragala‐Pinkham , N. Lennon , and M. E. O'neil . 2016. “Decision Trees for Detection of Activity Intensity in Youth With Cerebral Palsy.” Medicine & Science in Sports & Exercise 48, no. 5: 958–966. 10.1249/MSS.0000000000000842. 26673127 PMC4833604

[ejsc12313-bib-0044] Valadão, P. , F. Cenni , H. Piitulainen , J. Avela , and T. Finni . 2024. “Effects of the EXECP Intervention on Motor Function, Muscle Strength, and Joint Flexibility in Individuals With Cerebral Palsy.” Medicine & Science in Sports & Exercise 56, no. 1: 1–12. 10.1249/MSS.0000000000003273. 37565430 PMC11805469

[ejsc12313-bib-0045] Valadão, P. , H. Piitulainen , E. A. Haapala , T. Parviainen , J. Avela , and T. Finni . 2021. “Exercise Intervention Protocol in Children and Young Adults With Cerebral Palsy: The Effects of Strength, Flexibility and Gait Training on Physical Performance, Neuromuscular Mechanisms and Cardiometabolic Risk Factors (EXECP).” BMC Sports Science, Medicine and Rehabilitation 13, no. 1: 17. 10.1186/s13102-021-00242-y. PMC7908003 33637124

[ejsc12313-bib-0046] van den Berg‐Emons, R. J. , J. B. Bussmann , and H. J. Stam . 2010. “Accelerometry‐Based Activity Spectrum in Persons With Chronic Physical Conditions.” Archives of Physical Medicine and Rehabilitation 91, no. 12: 1856–1861. 10.1016/j.apmr.2010.08.018. 21112426

[ejsc12313-bib-0047] van den Hecke, A. , C. Malghem , A. Renders , C. Detrembleur , S. Palumbo , and T. M. Lejeune . 2007. “Mechanical Work, Energetic Cost, and Gait Efficiency in Children With Cerebral Palsy.” Journal of Pediatric Orthopaedics 27, no. 6: 643. 10.1097/BPO.0b013e318093f4c3. 17717464

[ejsc12313-bib-0048] van Sluijs, E. M. F. , U. Ekelund , I. Crochemore‐Silva , et al. 2021. “Physical Activity Behaviours in Adolescence: Current Evidence and Opportunities for Intervention.” Lancet 398, no. 10298: 429–442. 10.1016/S0140-6736(21)01259-9. 34302767 PMC7612669

[ejsc12313-bib-0049] Verschuren, O. , M. D. Peterson , A. C. J. Balemans , and E. A. Hurvitz . 2016. “Exercise and Physical Activity Recommendations for People With Cerebral Palsy.” Developmental Medicine and Child Neurology 58, no. 8: 798–808. 10.1111/dmcn.13053. 26853808 PMC4942358

[ejsc12313-bib-0050] Waltersson, L. , and E. Rodby‐Bousquet . 2017. “Physical Activity in Adolescents and Young Adults With Cerebral Palsy.” BioMed Research International 2017: 1–6. 10.1155/2017/8080473. PMC5750505 29423412

[ejsc12313-bib-0051] Yoon, M.‐J. , H. Choi , J.‐S. Kim , S. H. Lim , Y.‐J. Yoo , and B. Y. Hong . 2022. “Physical Activity, Quality of Life and Parenting Stress in Children With Cerebral Palsy.” Pediatrics International: Official Journal of the Japan Pediatric Society 64, no. 1: e15295. 10.1111/ped.15295. 36112040

